# Placenta Percreta Previa Crossing Surgical Frontiers

**DOI:** 10.7759/cureus.85028

**Published:** 2025-05-29

**Authors:** Chrysoula Margioula-Siarkou, Stefanos Flindris, Christina Yfanti, Elif Empliouk, Stamatia Angelidou, Fotios Chatzinikolaou, Eleni Mouloudi, Georgios Mavromatidis, Alexandros Sotiriadis, Konstantinos Dinas, Stamatios Petousis

**Affiliations:** 1 2nd Department of Obstetrics and Gynecology, Aristotle University of Thessaloniki School of Medicine, Thessaloniki, GRC; 2 Department of Pathology, General Hospital of Thessaloniki Ippokratio, Thessaloniki, GRC; 3 Laboratory of Forensic Medicine &amp; Toxicology, Aristotle University of Thessaloniki, Thessaloniki, GRC; 4 Department of Adult Intensive Care Unit, Ippokratio General Hospital, Thessaloniki, GRC; 5 2nd Department of Obstetrics and Gynecology, Ippokrateio Hospital of Thessaloniki, Thessaloniki, GRC

**Keywords:** cesarean hysterectomy, icu, parametria, placenta percreta, placenta previa

## Abstract

Placenta percreta represents a severe form of placenta accreta spectrum (PAS), characterized by the full-thickness invasion of placental villi through the uterine wall, often extending into adjacent structures. Its incidence is rising in parallel with the increased rates of cesarean sections and uterine interventions.

We report a case involving a patient 37^+6 ^weeks of gestation with a history of four cesarean sections and inadequate prenatal surveillance that was admitted with symptoms of labor onset. Intraoperatively, the placenta was found to invade not only the myometrium but also the parametrial tissue and uterine arteries, complicating the surgical field. An emergency cesarean section was performed, resulting in the delivery of a neonate in excellent health. However, due to massive hemorrhage and extensive local invasion, the patient required a radical hysterectomy with bilateral salpingo-oophorectomy, alongside multiple reoperations to address ensuing complications such as hemoperitoneum and uroperitoneum.

The complexity of the case necessitated prompt multidisciplinary intervention, including hemostatic techniques, urological interventions and vigilant perioperative care. Administration to ICU, multidisciplinary management, encompassing renal support, urinary bladder repair, temporary nephrostomies, management of hospital infections, structured physiotherapy and speech therapy were crucial in stabilizing the patient and facilitating gradual recovery.

Placenta percreta with parametrial and uterine artery invasion is a challenging clinical scenario that demands early diagnosis and coordinated multidisciplinary management. Optimizing prenatal screening protocols and surgical preparedness is essential to mitigate the high risks of maternal morbidity and mortality associated with this condition.

## Introduction

Placenta accreta spectrum (PAS) disorders represent a group of abnormal placental implantations characterized by varying degrees of invasion into the uterine wall [1]. At one end of this spectrum is placenta accreta, where the placenta attaches superficially to the myometrium without true invasion, while placenta increta involves partial penetration through the myometrium [2]. The most severe form, placenta percreta, is defined by placental villi invading through the entire uterine wall, breaching the serosa, and potentially extending into adjacent pelvic or abdominal organs [3]. This spectrum is of increasing clinical significance due to the rising incidence of cesarean sections and other uterine interventions (curettage, scars, Asherman's syndrome, etc.), which contribute to defective decidualization and predispose women to these life‐threatening conditions [1,4].

The severity of PAS can be determined at the time of laparotomy using the International Federation of Gynecology and Obstetrics (FIGO) grading system, which classifies PAS into the three categories. Grade 1 (G1) indicates the abnormally adherent placenta (placenta adherenta or creta), G2 reflects the abnormally invasive placenta (increta) and G3 denotes the abnormally invasive placenta (percreta). G3 is further subdivided into the G3a when invasion is limited to the uterine serosa, G3b when the urinary bladder is involved and G3c when other pelvic tissues or organs are invaded [5]. The placental bulk often occupies the narrow confines of the pelvis, complicating the identification of vital anatomical structures such as the distal ureters and uterine arteries [5]. Moreover, abnormal neovascularization and distorted anatomical planes, especially in patients with prior cesarean sections, further increase the risk of intraoperative bleeding and collateral damage to surrounding organs [5,6].

Placenta percreta carries substantial maternal morbidity and mortality, with hemorrhage-related complications in up to 7-10% of cases and overall complication rates approaching 60% [7]. Its incidence has increased tenfold over the past 50 years and now occurs at a frequency of approximately 1 per 2,500 deliveries [6,7]. The highest incidence was observed in lower middle-income settings (3/1,000 births, 95% CI 2.5-3.5), and the lowest incidence was observed in high-income settings (0.7/1,000 births, 95% CI 0.5-0.8) [8]. This disorder confers an exceptionally high risk of massive hemorrhage, which may precipitate multisystem organ failure and disseminated intravascular coagulation (DIC) and, when extrauterine invasion is confirmed, cesarean hysterectomy with the placenta left in situ remains the definitive surgical approach.

When placenta percreta is suspected antenatally, current FIGO and American College of Obstetricians and Gynecologists (ACOG) international guidelines recommend delivery at 34 0/7-35 6/7 weeks in a tertiary center equipped with multidisciplinary surgical, anesthesia, interventional radiology, and blood-bank teams to optimize outcomes [4,6,7,9]. In women with multiple prior cesarean deliveries, targeted screening for PAS, beginning with detailed ultrasound, and, where indicated, supplemented by MRI, is strongly recommended early in the third trimester to facilitate timely referral and surgical planning. Nonetheless, unexpected intraoperative findings still pose formidable challenges, underscoring the critical need for early diagnosis and comprehensive peripartum planning [4,6].

This grading is critical not only for prognostication but also for tailoring surgical management. Clinically, placenta percreta is associated with significant maternal morbidity and mortality [2]. The condition poses a high risk of massive hemorrhage, potentially leading to multisystem organ failure and DIC [10]. In severe cases, where extrauterine invasion is confirmed intraoperatively, cesarean hysterectomy remains the definitive management strategy [5]. However, the surgical challenges are formidable [2].

The present case report focuses on a patient with placenta percreta, underscoring the complexities associated with extrauterine invasion. Improved prenatal detection, timely referral to specialized centers, and the implementation of adjunct hemostatic measures are paramount in reducing surgical morbidity in these high-risk patients.

## Case presentation

A woman in her 20s, Gravida 5 Para 4 (G5P4), at 37 weeks of gestation with a history of four previous cesarean sections (PCSs), was admitted to the emergency department of the 2nd Department of Obstetrics and Gynecology, General Hospital of Thessaloniki Ippokratio, late in the evening, presenting with acute abdominal pain. Notably, the patient had not undergone any scheduled antenatal examinations or surveillance during this pregnancy. Her fourth cesarean delivery had been performed two years prior, marking her fourth consecutive cesarean section. She had no history of abortion or other abdominal surgeries.

On initial examination, there was no vaginal bleeding. Her vital signs were stable, with a pulse of 86 beats per minute and a blood pressure of 104/62 mmHg. The Glasgow Coma Scale (GCS) score was 15/15. The hemoglobin (Hb) was 11.4 g/dL, hematocrit (Hct) was 34.7%, platelets were 140.000/μL, prothrombin time (PT) 11.1 sec, International Normalized Ratio (INR) 1.01, activated partial thromboplastin time (APTT) 28.6 sec, and fibrinogen 431.7 mg/dL. Cardiotocography demonstrated a normal fetal heart rate with frequent, painful uterine contractions. Routine antenatal ultrasound revealed an anterior placenta covering slightly the internal cervical os. Clinical examination elicited significant tenderness and pain localized to the PCS scar site. It should be noted that MRI, often used to further delineate placental invasion, was not feasible in this emergency setting, and management decisions relied upon high-resolution sonography.

Given these findings, an emergency cesarean section was performed under epidural anesthesia on day zero. A female infant weighing 2,800 g was delivered in breech presentation, with Apgar scores of 8 and 9 at one and five minutes, respectively. During laparotomy via a Pfannenstiel incision, the surgical team identified large vessels traversing the anterior uterine wall. The urinary bladder was found to be densely adherent to the anterior uterine surface, extending laterally to the level of the round ligaments. A small tear was observed in the lower lateral anterior uterine wall, posing a risk of uterine rupture. Consequently, the neonate was delivered via a transverse uterine incision made superior to the bladder’s attachment.

Shortly after manual removal of the placenta, the patient developed profuse hemorrhage, revealing a large uterine defect on the anterior surface. Due to uncontrolled bleeding, conversion to an obstetric hysterectomy was required. Intraoperatively, the surrounding tissues were extremely fragile and hemorrhagic. To optimize surgical exposure, the Pfannenstiel incision was extended medially and vertically to the umbilicus. A radical abdominal hysterectomy (Type C per the Querleu-Morrow classification) with bilateral salpingo-oophorectomy was performed due to persistent hemorrhage originating from the anterior and lateral parametrial regions and the mesosalpinx. Hemostatic agents, including Surgicel Powder and Veriset, were utilized to manage diffuse bleeding from the ligated parametrial tissues and the vaginal cuff, likely secondary to DIC.

Histopathological examination of the resected uterus, parametria, adnexa, and anterior cervical os confirmed the presence of trophoblastic tissue consistent with placenta percreta. Additionally, intraoperative bladder injury, resulting from morbid placental adherence and invasion extending to the uterine fundus, necessitated repair by a urologist using a two-layer closure technique. The uterus, parametria, adnexa, placenta, and a segment of the upper vagina were submitted for further histopathological evaluation.

During the procedure, the patient experienced two episodes of intraoperative cardiac arrest secondary to hemorrhagic shock; prompt advanced life-support measures successfully restored cardiovascular function. The estimated blood loss exceeded 4,000 mL. To maintain hemodynamic stability, she received crystalloids, 10 units of packed red blood cells, eight units of fresh-frozen plasma, three units of platelets, tranexamic acid, fibrinogen concentrate, prothrombin complex concentrate, sodium bicarbonate, and calcium chloride. In such critical scenarios, rotational thromboelastometry (ROTEM) provided real-time assessment of coagulation dynamics, including clot formation and fibrinolysis, and guided targeted transfusion and hemostatic therapy. Other potential interventions include placement of a resuscitative endovascular balloon occlusion of the aorta (REBOA) under angiography in an elective setting (not available at our institution) or ligation of the anterior branch of the internal iliac artery. The former was not available, and the latter was initially avoided due to the risk of iatrogenic injury to adjacent large vessels but would be considered if hemorrhage persisted.

**Figure 1:**
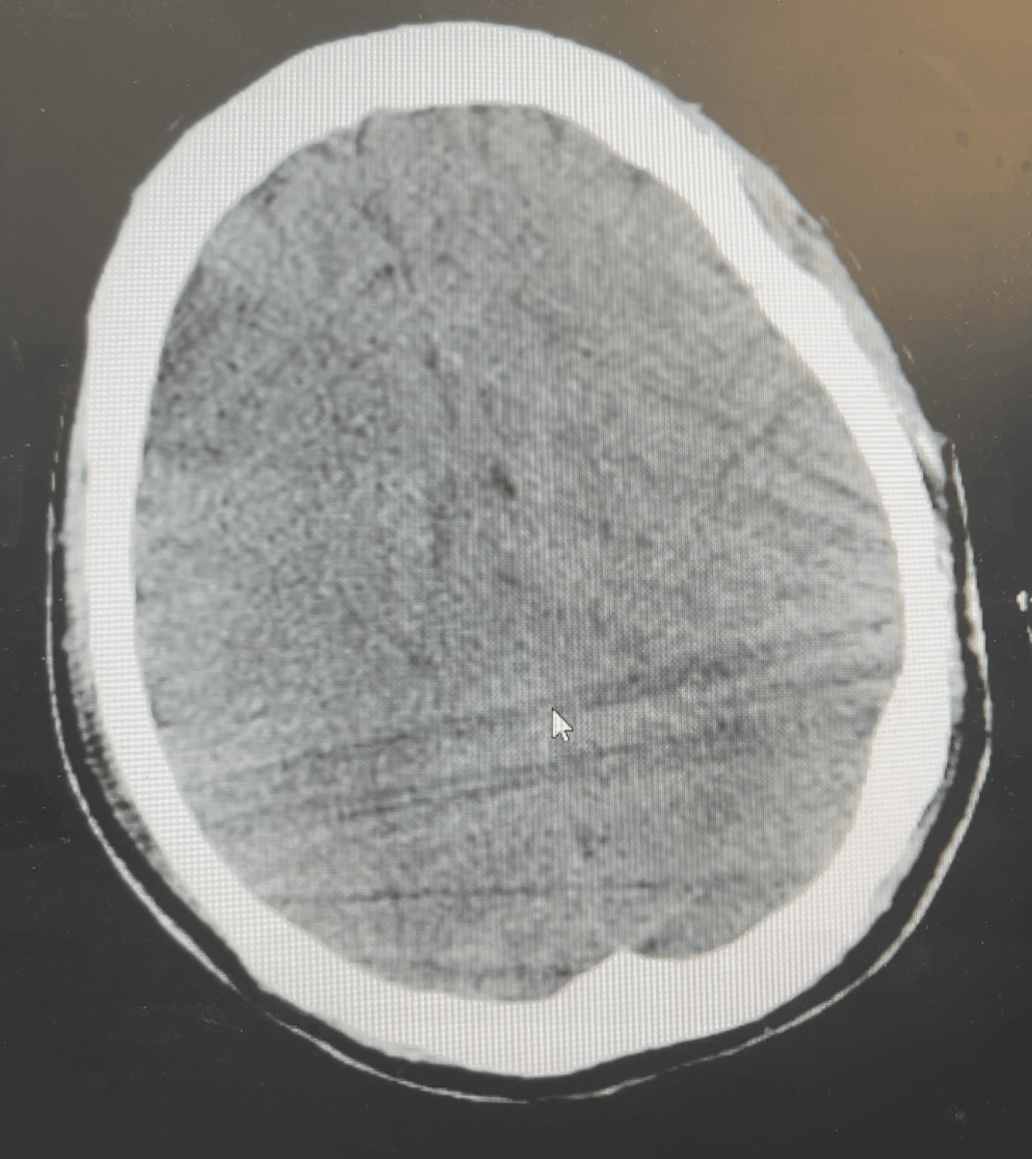
Axial non-contrast CT of the brain demonstrating diffuse cerebral hypoattenuation with loss of gray-white matter differentiation and sulcal effacement, consistent with global cerebral edema in the post-cardiac arrest setting.

Postoperatively, the patient was admitted to the ICU for close monitoring while intubated and hemodynamically supported (Hb 6.5 g/dL, Hct 19.8%). Lactation suppression was achieved with lisuride 75 mg once daily for 10 days. Despite aggressive fluid resuscitation and transfusion of blood products, she developed acute renal failure, necessitating initiation of continuous venovenous hemodiafiltration (CVVHDF). On postoperative day two, a full-body CT scan revealed cerebral edema and areas suggestive of ischemic stroke, likely secondary to the intraoperative cardiac arrests and DIC (Figure 1). The neurology and neurosurgery teams managed these complications, placing an intracranial pressure, monitoring and drainage device to maintain stable intracranial pressure and minimize fluctuations. Concurrently, she was diagnosed with adrenal insufficiency secondary to hemorrhagic shock. Hydrocortisone replacement therapy was initiated immediately, with additional hormonal supplementation begun 15 days postoperatively.

**Figure 2:**
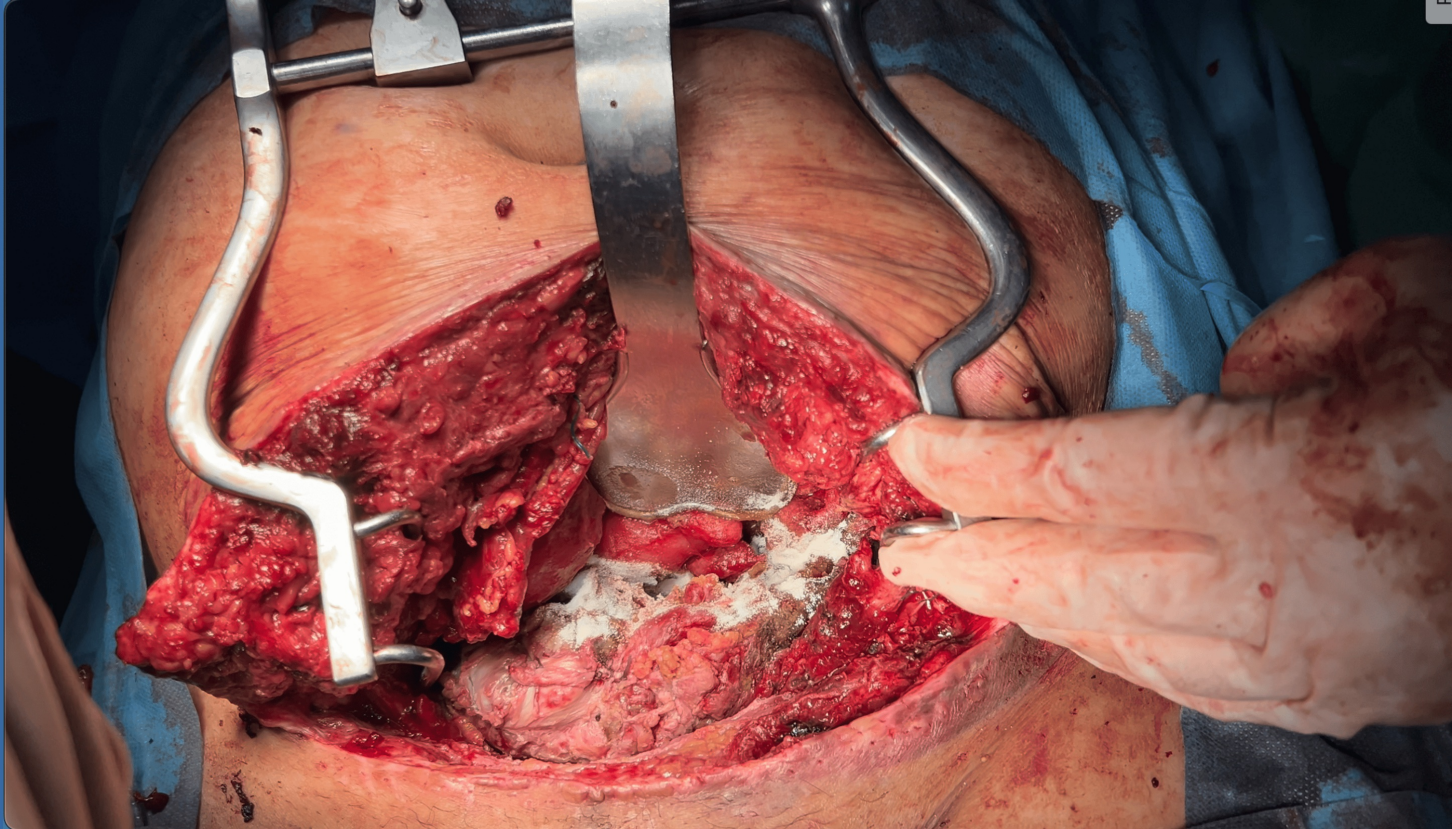
The intraoperative image demonstrates complete drainage of the hemoperitoneum, successful hemostasis and application of hemostatic powder

On day seven after the initial surgery, signs of urinary leakage and uroperitoneum were confirmed by CT intravenous urography, necessitating a subsequent abdominal surgery for bladder repair. On day nine, an embolus was detected in the inferior vena cava, prompting initiation of low-molecular-weight heparin (LMWH) for anticoagulation. However, on postoperattive day nine after starting LMWH, the patient developed hemostatic instability, manifesting as hemorrhage from catheter insertion sites and the vaginal cuff. This was followed by further hemodynamic deterioration and intraperitoneal hemorrhage, necessitating an emergency laparotomy for hemostatic control (Figure 2).

**Figure 3:**
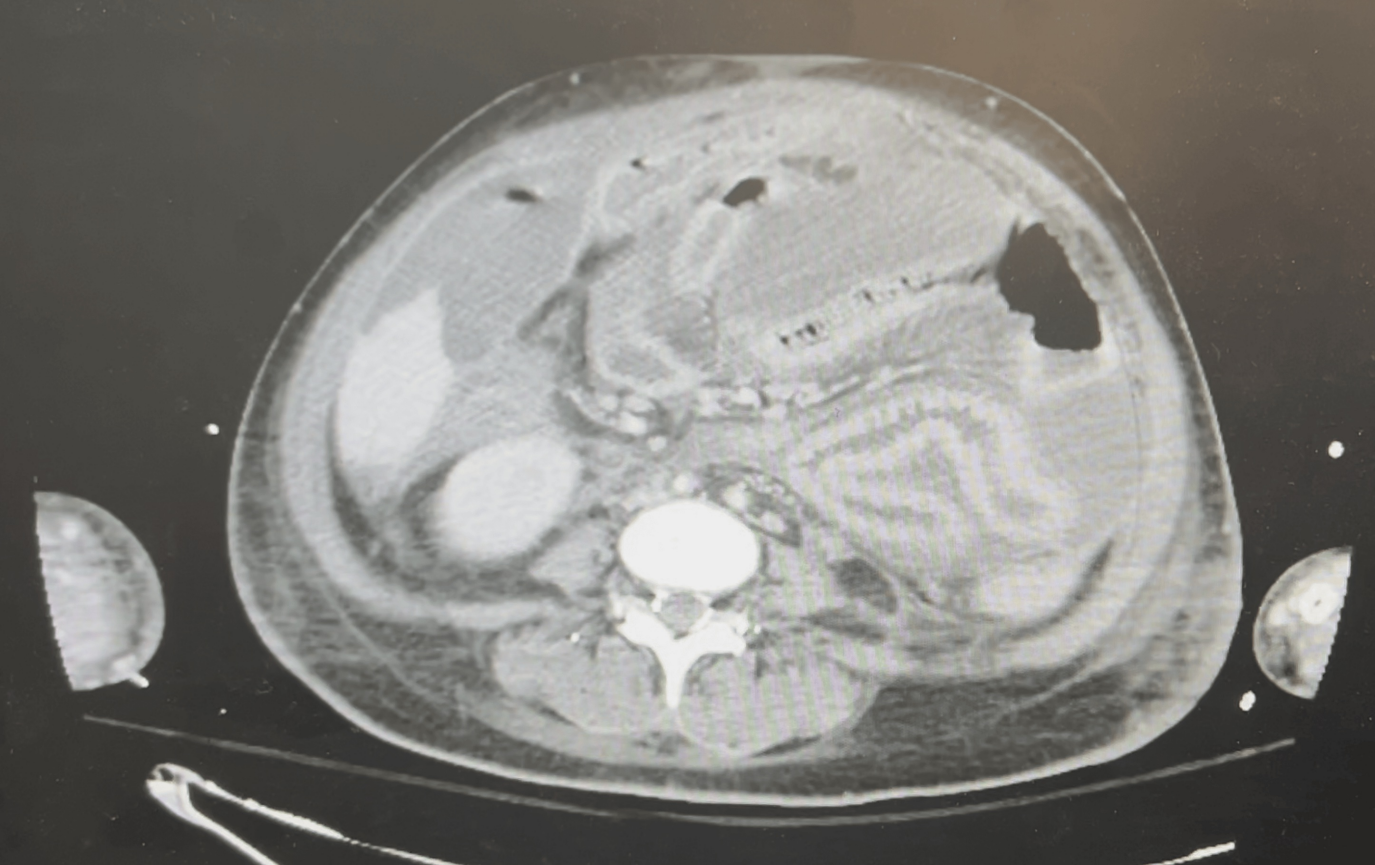
Abdominal CT scan demonstrating large intra-abdominal fluid collections, likely consistent with uroperitoneum, indicative of uroperitonitis

**Figure 4:**
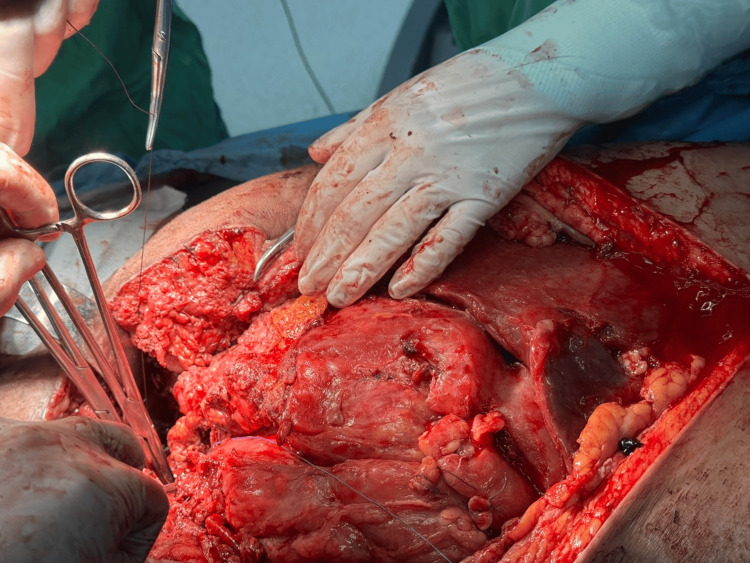
Intraoperative image of midline laparotomy denotes the plastron of small, large bowel and omentum

**Figure 5:**
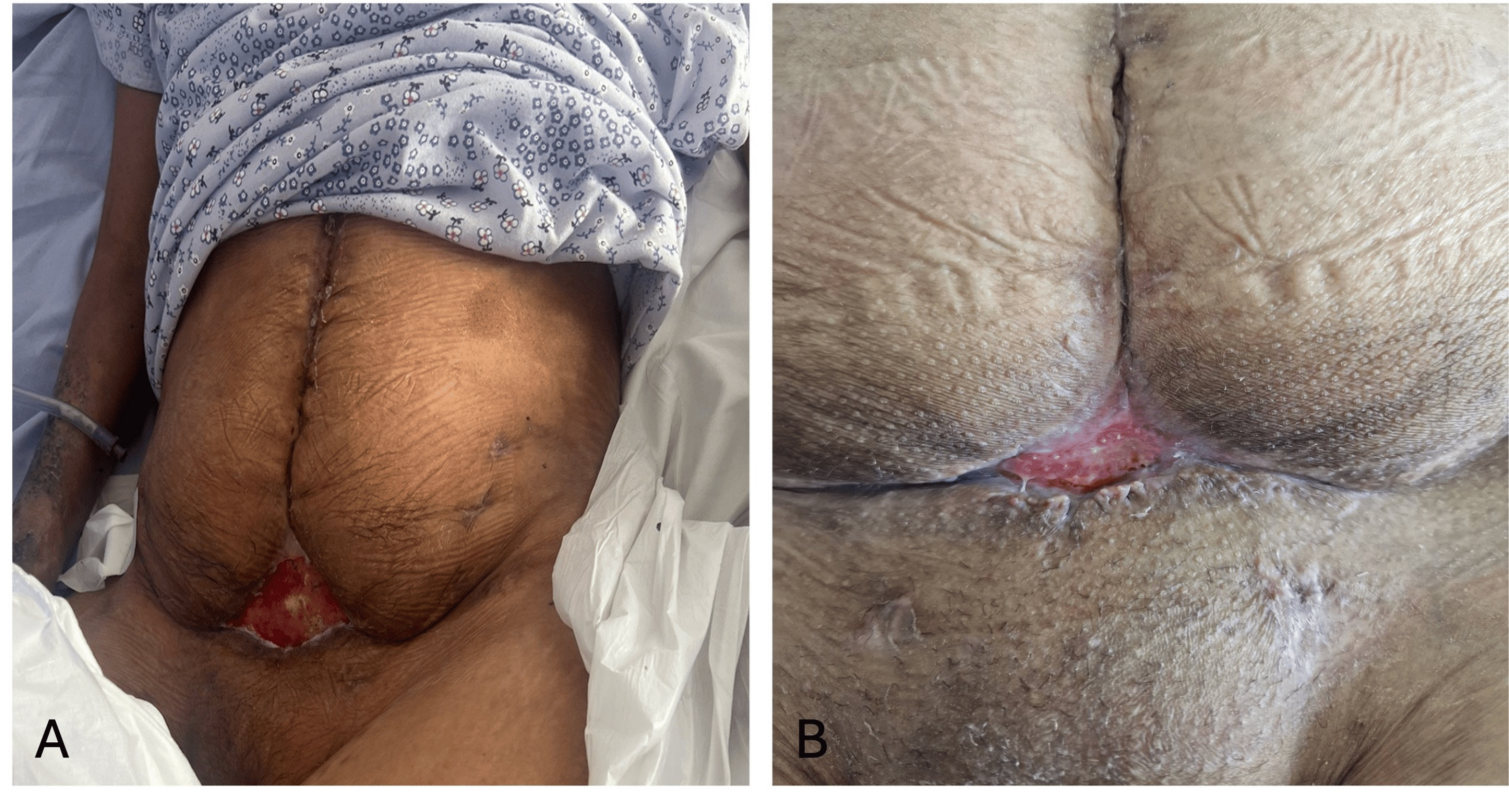
(A-B) Gradual surgical wound closure using VAC VAC: Vacuum-assisted closure

On day 20 postoperatively, an abdominal CT scan revealed large intra-abdominal fluid collections (Figure 3). An emergent surgical intervention was performed, revealing organized collections with plastron formation involving both the small and large intestines (Figure 4). Fluid analysis confirmed the presence of urine due to persistent bladder leakage, leading to a diagnosis of chemical peritonitis. Extensive abdominal lavage was performed, and four surgical drains were placed in each quadrant of the abdomen. Although initial attempts at CT-guided drainage were made, the patient's septic condition progressed, necessitating further surgical drainage on day 27. Ultimately, due to the inability to preserve bladder integrity, the urology team placed bilateral nephrostomies on day 30. After this fifth reoperation, a vacuum-assisted closure (VAC) device was employed for secondary trauma closure (Figure 5).

Following these complex interventions, the patient remained in the ICU for three months. She was subsequently transferred to the 2nd Department of Obstetrics and Gynecology at the General Hospital of Thessaloniki Ippokratio for continued management, rehabilitation, and follow-up. By the time of transfer, her renal function had returned to physiological levels, allowing discontinuation of CVVHDF, and both nephrostomies were removed. She then underwent intensive physiotherapy five times weekly, including standing practice and balance training that progressed from parallel bars to walker-assisted standing, ultimately achieving a Berg Balance Scale score of 42/56. Daily speech therapy sessions employed Frenchay Dysarthria Assessment-2 (FDA-2)-guided drills (diadochokinesis exercises, phonation control) and Gugging Swallowing Screen (GUSS)-recommended swallowing maneuvers (chin-tuck, effortful swallow). Motor function began to recover in both upper and lower extremities. Intensive physiotherapy played a crucial role in functional rehabilitation, stabilization of septic parameters, maintenance of hemodynamic stability, and progressive neurological improvement.

A neurological examination conducted a few days before discharge revealed a slight hemiparesis of the right hand and the patient was alert and oriented with a GCS of 15/15 and the modified Rankin Scale (mRS) was 3. During her hospital stay, she received a total of 72 units of packed red blood cells. The neonate remained in excellent health, showing no signs of complications. The patient was discharged on day 178 after admission (six months hospitalization), demonstrating remarkable recovery (Hb 11.8 g/dL and Hct 37.6%), standing almost independently and initiating ambulation with support from assistive devices or family members.

At the four-week follow-up after discharge, her neurological exam demonstrated a National Institutes of Health Stroke Scale (NIHSS) score of 2, reflecting only a mild right-hand drift, and Medical Research Council (MRC) grading showed 4+/5 strength in the right hand with full strength elsewhere. Her Berg Balance Scale score rose to 45/56, allowing her to stand unsupported for more than two minutes and perform independent weight shifts. On the Functional Independence Measure (FIM), she required only minimal assistance for transfers and was able to ambulate 25 m with a quad cane. Speech assessment (FDA-2) was entirely normal, with 100% intelligibility, and swallowing evaluation (GUSS) confirmed safe oral intake on a regular diet using compensatory maneuvers. Formal re-evaluations of her standing and ambulation are planned at three months, including the Berg Balance Scale to quantify balance and the mRS to assess global disability and functional independence. A multidisciplinary clinic review at six months will integrate these results to optimize her ongoing rehabilitation plan.

Histopathological examination

**Figure 6:**
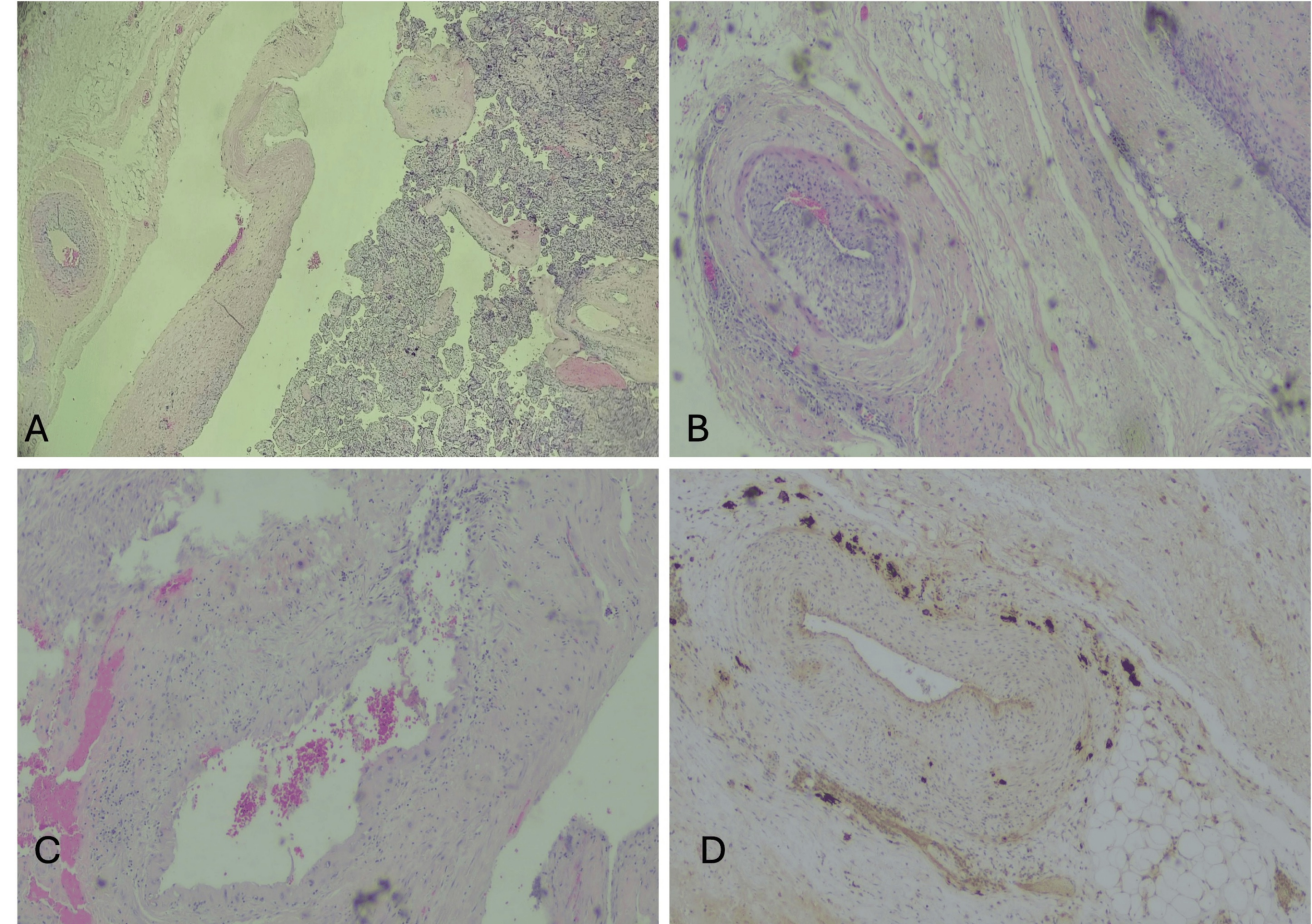
(A) Placenta infiltration towards adipose tissue of adjacent parametria, disturbed architecture of basal plate that is in connection with chorionic villi in the upper part and absence of intervening decidual cells; (B) demonstrates a branch of the uterine artery within the parametrium infiltrated by trophoblastic cells; (C) depicts a branch of the uterine artery in the cervix with trophoblastic cells; (D) demonstrates positive IHC expression for AE1/AE3 in trophoblastic cells infiltrating the wall of a uterine artery branch IHC: Immunohistochemistry

Microscopic evaluation confirmed a diagnosis of placenta percreta (PAS, FIGO G3C), characterized by placental villi invading the parametrial tissue and adjacent adipose structures (Figure 6A). There was complete disruption of the basal plate with an absence of intervening decidual cells. Scattered multinucleated extravillous trophoblastic cells were observed infiltrating both the parametria and maternal uterine arteries with concentric thickness of their wall and transmural invasion of extravillous trophoblastic cells extending into the subadventitial layers of the cervix (Figures 6B, 6C). Immunohistochemical staining for pan-cytokeratin (AE1/AE3) confirmed the trophoblastic origin of these infiltrating cells (Figure 6D).

Additional histopathological findings included microglandular hyperplasia of the endocervical epithelium and pronounced decidualization of the cervical stroma, consisting of huge, polygonal-shaped, pale pink stroma cells, are morphological characteristics confirming the presence of pregnancy. Further supporting the diagnosis of deep parametrial and cervical invasion by abnormal placental tissue [2].

## Discussion

The main objective of the present case report was to describe the demanding case of a plcenta percreta invading parametria and uterine artery, necessitating radical surgical approach, resuscitation after cardiac arrest and multiple reinterventions to provide optimal treatment.

Placenta percreta is a rare obstetric complication that can lead to disastrous outcomes due to massive hemorrhage [11,12]. Risk factors include the number of PCSs, with a risk ratio of 40% for two PCSs and increasing with each additional cesarean [6]. Other risk factors include uterine curettage and uterine scars from other gynecological surgeries, such as myomectomy [11]. It is of great importance that the diagnosis is made before 30 weeks of gestation, allowing for appropriate multidisciplinary management and comprehensive preparation, ideally led by a gynecological oncologist surgeon (including blood banks, vascular surgeons, etc.) in an elective procedure before 35 weeks [13,14].

Our case further illustrates that once PAS is suspected or diagnosed, whether in a scheduled or emergent setting, a dedicated multidisciplinary team (obstetricians, anesthesiologists, gynecologic oncologists, urologists, general surgeons, vascular surgeons and critical care specialists) must be mobilized without delay. Preoperative placement of ureteric stents, availability of cell-saver technology, and predefined hemorrhage protocols can mitigate catastrophic hemorrhage and optimize maternal outcomes [15].

The gold-standard management of PAS disorders is obstetric hysterectomy, ideally without removing the placenta, and the uterine incision should be made far from the edge of the placenta [6]. In very rare cases, like the one reported here, placental invasion may extend deep into the parametria, necessitating more radical hysterectomy techniques, including internal iliac artery ligation or even resuscitative aortic compression to control hemorrhage [3,13]. However, some authors have described uterus-conserving techniques, which can only be applied in selected patients and at highly specialized centers [16,17].

In contrast to the urgent, life-threatening hemorrhage mandates of emergency PAS surgery, many experts advocate for a scheduled, preterm cesarean hysterectomy with the placenta left in situ to ensure optimal preparation and resource allocation. The ACOG Obstetric Care Consensus recommends delivery at 34-35 weeks by a multidisciplinary team, with the placenta left undisturbed to minimize blood loss and organ injury [18]. In a single-center Canadian cohort of 125 PAS cases, electively scheduled cesarean-hysterectomy was associated with significantly lower estimated blood loss (1,561 ± 1,153 mL vs. 2,772 ± 2,257 mL), rates of coagulopathy (6% vs. 40%), and bladder injury (13% vs. 44%) compared with emergency deliveries [19]. Moreover, when invasive percreta is diagnosed antenatally, an interval (delayed) hysterectomy performed four to six weeks after initial cesarean delivery has been shown to halve cumulative blood loss (1,300 mL vs. 3,000 mL) and reduce transfusion needs relative to immediate hysterectomy [20].

The basis for diagnosis is ultrasound in the second or third trimester, though the diagnostic accuracy is substantially lower when performed by inexperienced practitioners, especially when the placenta is not previa [1,5,13]. MRI can assist with differential diagnosis and effectively depict the extent of invasion, but the final diagnosis is typically made intraoperatively, based on FIGO classification [5,11]. Ultrasound findings, such as multiple vascular lacunae within the placenta, loss of the hypoechoic zone between the placenta and myometrium near the bladder, irregular bladder walls, hypervascularization, and loss of the myometrial interface, are suggestive of PAS and these features also appear on MRI [21,22].

In line with the FIGO 2018 consensus guidelines, a standardized prenatal imaging protocol, including targeted ultrasound assessment of myometrial thickness and adjunctive MRI for suspected increta or percreta is recommended to grade invasion accurately and guide management [9]. The ACOG Obstetric Care Consensus No. 7 further underscores a multidisciplinary approach, advising preoperative planning with MRI mapping and prophylactic ureteric stent placement to minimize urinary tract injury [7]. Furthermore, a recent US national cohort review of placenta percreta with adjacent-organ extension, including the bladder, cervix, and parametria, reported severe maternal morbidity in 82.1% of cases and a mortality rate of 1.4% [23]. Also, Palacios-Jaraquemada et al. reported that lower-segment and parametrium invasion by PAS is relatively uncommon, but when parametrial involvement does occur, it is associated with markedly increased intraoperative blood loss, greater transfusion requirements, and extended operative times, driving maternal morbidity rates to over 80% [24]. Those studies highlight the particularly high risks when parametrial invasion is present and reinforcing the need for meticulous preoperative diagnosis and surgical planning. In our department, the standard protocol includes preoperative MRI to confirm the diagnosis and the placement of ureteric stents a few days before the elective operation as other centers do respectively [3,14].

In the current case, the diagnosis of placenta percreta was made intraoperatively during an emergent cesarean section in labor, performed under epidural analgesia, which is far from the ideal approach in these situations. This case underscores the relevance of early diagnosis and adequate surgical preparation, including necessary resources such as Bookwalter or Omnitract auto retractors. Furthermore, it illustrates the importance of suspicion in women with three or more PCSs who lack proper prenatal surveillance and the importance of radical surgery specialization [13].

## Conclusions

Placenta percreta, particularly when extending into parametrial tissue and uterine arteries, represents one of the most formidable challenges in obstetric surgery, demanding anticipatory planning, precise imaging, and rapid multidisciplinary coordination to optimize maternal outcomes. This case underscores the necessity of high clinical suspicion in patients with multiple prior cesarean deliveries and inadequate prenatal surveillance, the critical role of advanced hemostatic and surgical techniques, including radical hysterectomy approaches, internal iliac artery ligation, or temporary vascular occlusion of the aorta just proximal to its bifurcation when conservative measures fail and the indispensable requirement for immediate, on-call availability of experienced gynecological oncologist surgeons alongside experienced anesthisiologists, vascular surgeons, urologists, and dedicated ICU teams. Equally vital is the establishment and regular rehearsal of institutional protocols for these high-risk scenarios, ensuring that every member of the care team from is fully prepared to act decisively under urgent and stressful conditions. Furthermore, intensive postoperative multidisciplinary support and tailored rehabilitation strategies are essential to secure recovery. Ultimately, proactive referral to specialized centers, coupled with thorough prenatal screening protocols, seamless team readiness, and robust institutional preparedness, can transform an otherwise catastrophic scenario into a survivable event, reaffirming that systematic planning and collective expertise are paramount to reducing the morbidity and mortality inherent in severe PAS disorders.
